# Comparative evaluation of HPV genotyping: A study on the performance concordance between Anyplex II HPV28 detection and Linear Array genotyping tests in nationwide studies in Brazil

**DOI:** 10.1371/journal.pone.0305122

**Published:** 2024-06-11

**Authors:** Isabel Cristina Bandeira, Juliana Comerlato, Marina Bessel, Bruna Vieira Fernandes, Giana Mota, Luisa Lina Villa, Flávia Moreno Alves de Souza, Gerson Fernando Mendes Pereira, Eliana Marcia Wendland

**Affiliations:** 1 Hospital Moinhos de Vento, Porto Alegre, Brazil; 2 Innovation in Cancer Laboratory, Center for Translational Research in Oncology of the Instituto do Câncer do Estado de São Paulo (ICESP), São Paulo, Brazil; 3 Department of Chronic Conditions and Sexually Transmitted Infections, Ministry of Health, Brasília, Brazil; 4 Department of Community Health, Universidade Federal de Ciências da Saúde de Porto Alegre, Porto Alegre, Brazil; Mie University Hospital: Mie Daigaku Igakubu Fuzoku Byoin, JAPAN

## Abstract

**Background:**

Advances in laboratory techniques for HPV diagnosis necessitate a thorough assessment of the efficiency, replicability, sensitivity, and specificity of those methods. This study aims to validate and compare HPV detection/genotyping using the Anyplex™ II HPV28 Detection assay (Seegene) assay and the Linear Array HPV Genotyping test (Roche Diagnostics) on genital samples for use in epidemiological studies.

**Methods:**

From 6,388 penile and cervical DNA samples collected in the POP-Brazil, 1,745 were randomly selected to be included in this study. The samples were submitted to HPV detection and genotyping following the manufacturers’ protocols. DNA was genotyped using the Anyplex™ II HPV28 Detection kit (Seegene), and the results were compared to those obtained using the Linear Array HPV Genotyping test (Roche Diagnostics). Concordance of HPV genotyping results was assessed by the percentage agreement and Cohen’s kappa score (κ).

**Results:**

The agreement between the two methodologies was deemed good for HPV detection (κ = 0.78). Notably, Anyplex™ II HPV28 demonstrated enhanced capability in detecting a broader spectrum of genotypes compared to Linear Array.

**Conclusion:**

Anyplex™ II HPV28 exhibited comparable results to the Linear Array assay in clinical specimens, showcasing its potential suitability for a diverse array of research applications requiring the detection and genotyping of HPV. The study supports the utility of Anyplex™ II HPV28 as an effective tool for HPV screening in epidemiological studies, emphasizing its robust performance in comparison to established diagnostic tests.

## Introduction

Human papillomavirus (HPV) is the most common sexually transmitted infection and a causative agent of virus-driven cancer. Most of the papillomaviruses cause asymptomatic infection; however, several types have evolved mechanisms to promote cellular transformation [1, 2]. Persistent infection with high-risk human papillomavirus (HR-HPV) is considered the most important risk factor for development of cervical cancer [3]. In 2020, worldwide estimates revealed that cervical cancer ranked as the fourth most prevalent cancer globally, with an approximate incidence rate of 13.3 cases per 100,000 women-years and a mortality rate of 7.2 deaths per 100,000 women-years.[4]. HPV is also accountable for a substantial proportion of cancers and precancerous lesions, not only in the head and neck but also in the genital regions, including penile, vulvar and anal cancers [5, 6].

HPV genotype testing represents an important tool for studying the carcinogenic potential of HPV types and is used widely to screen for and follow-up persistent infections that could lead to cervical cancer [7, 8]. In epidemiology, HPV assays are mainly used to determine the prevalence of HPV types distributed in the study population [9, 10]. Nowadays, more than 250 assays are available to detect and/or genotype HPV [11]. Although tests restricted to identification of the more prevalent HR-HPV types can be used in triage, epidemiological studies generally need to know a broad number of HPV types in order to evaluate the type distribution in the population, the impact of vaccination, and cross-protection. Additionally, HPV genotyping techniques need to be evaluated over time using the same or equivalent tests to avoid biases. The Linear Array® HPV Genotyping test (Roche Diagnostics) (LA) was considered the gold standard for many years and was also used in research on HPV epidemiology, cervical cancer screening, and vaccine surveillance [12, 13]. The LA applies a reverse hybridization assay based on the L1 gene PGMY09/11 consensus primer set [14]. This assay is capable of detecting 37 genotypes and consists of a PCR followed by in situ hybridization and subsequent reading on a nylon strip coated with HPV type-specific and human beta-globin-specific oligonucleotide probes [15]. However, the LA was discontinued in December 2019, which made it necessary to evaluate new available methodologies [16, 17]. Over the years, several new diagnostic methodologies that seek greater efficiency have been implemented and have gained prominence in the market, which currently has more than 250 commercial tests for HPV diagnosis that vary in methodologies, genotype coverage, feasibility, and costs [12, 18].

Among the available DNA-based HPV detection and genotyping methods, the Anyplex™ II HPV28 Detection assay is a qualitative, multiplex, real-time polymerase chain reaction (RT-PCR) assay designed to detect low and HR-HPV types in two multiplex reactions targeting the L1 HPV region and has been an option since 2012. A region from the human beta-globin gene is co-amplified as an internal control to monitor DNA quality [9, 19].

For evaluating the performance of HPV tests, diverse and reliable external quality control programs based on international standards are essential. Aiming to compare and improve the guidelines of HPV assays clinically validated for high-risk HPV in cervical cancer screening, the VALGENT protocol was created. This protocol accounts with an established panel of cervical samples with abnormalities in cytology analyzed by different laboratories and methods to generate accuracy and concordance statistics [20, 21]. Simultaneously, the WHO global reference laboratory offers, each year, the “Global HPV DNA Proficiency Study” to certify laboratories as proficient in the genotyping of the main HPV types [22]. Both proficiency panels are composed of a series of validated and traceable samples of international standards in terms of generating acceptable results [23].

The aim of this study was to compare two HPV DNA-based genotyping methodologies, the Anyplex^**TM**^ II HPV28 Detection assay (Seegene) and the Linear Array® HPV Genotyping test (Roche Diagnostics), to evaluate the possibility of replacing the previous LA methodology in epidemiological studies.

## Methods

### Population/POP study

Study participants were recruited between September 2016 and November 2017 by the POP Brazil study, a nationwide, cross-sectional study designed to estimate the prevalence of type-specific HPV in young adults aged between 16 and 25, from both sexes and to serve as a baseline to assess the impact of the Brazilian national vaccination program [24]. Among the 6,388 penile and cervical DNA samples collected in the POP-Brazil study, 1,745 were randomly selected for this investigation.

### Sample collection and processing

The total of cervical and penile samples was collected, processed and submitted to HPV detection and genotyping using the LA assay as described in the POP-Brazil study [24]. Briefly, cervical samples were collected by a trained health professional using the Qiagen HC2 DNA collection device. Samples from men were self-collected from the entire surface of the scrotum, glans penis/coronal sulcus, and penile shaft using a saline-wetted Dacron swab [25, 26]. Both FMUSP and Clinical Epidemiology Laboratory of UFCSPA performed the HPV DNA typing proficiency study from Equalis, an external quality assessment for laboratories that perform HPV typing, organized by the Global HPV LabNet, to both HPV DNA assays included in this manuscript.

Following manufacturers’ protocols, the samples underwent HPV detection and genotyping using the Anyplex™ II HPV28 Detection kit (Seegene). The obtained results were then compared to those from the Linear Array HPV Genotyping test (Roche Diagnostics). This multiplex PCR assay simultaneously detects 19 high-risk HPV types and 9 low-risk HPV types using tagging oligonucleotide cleavage and extension™ (TOCE™) technology. Anyplex™ II HPV28 was chosen to contemplate the mainly HPV types included in the LA assay and also the most prevalent HPV types in the Brazilian population. Table 1 shows the genotypes covered by each assay compared in this study. The reactions were performed according to the manufacturers’ instructions.

**Table 1:** Genotypes covered by LA and Anyplex™ II HPV28. Genotypes in bold are exclusive to each test.

Linear Array	Anyplex™ II HPV28
6, 11, 16, 18, 26, 31, 33, 35, 39, 40, 42, 45, 51, 52, 53, 54, **55**, 56, 58, 59, 61, **62**, **64**, 66, **67**, 68, 69, 70, **71**, **72**, 73 (MM9), **81**, 82 (MM4), **83** (MM7), **84** (MM8), 82 (IS39), **89** (CP6108)	16, 18, 26, 31, 33, 35, 39, 45, 51, 52, 53, 56, 58, 59, 66, 68, 69, 73, 82, 6, 11, 40, 42, **43**, **44**, 54, 61, 70

We consider the samples as adequate when the internal control signal was observed, according to Anyplex™ II HPV28 and LA manufacturer’s instructions.

### Statistical analysis

Since each HPV type has a different occurrence rate, the draw was made so that the types with the lowest occurrence were not under sampled, resulting in an equivalent number of samples for each type. Concordance of HPV genotyping results was evaluated using the percentage agreement and Cohen’s score kappa (κ), which was classified as follows: 0.0 to 0.19, poor, 0.20 to 0.39, fair; 0.40 to 0.59, moderate; 0.60 to 0.79, good; 0.80 to 1.0, excellent [27]. The Anyplex™ II HPV28 Detection assay and LA were also evaluated for sensitivity and specificity. Simple agreement was calculated by adding the agreement of the positive and negative results for both techniques. The McNemar test was used to assess discordance (no shared genotypes). Statistical analysis was performed using SAS software (Statistical Analysis System, SAS Institute Inc., Cary, N.C.), version 9.4.

### Ethical aspects

This study was approved by the ethics review committees of all participating centers. Recruited individuals agreed voluntarily to participate and signed an informed consent form. The study follows the ethical recommendations of Resolutions 347/05, 441/11, and 466/12 of the National Health Council.

## Results

Of the 1,745 samples processed, 1,709 were suitable for analysis. Thirty-six samples provided inadequate results due to the absence of internal control amplification in the Anyplex™ II HPV28 assay, and 18 samples did not have a sufficient volume to be processed.

The overall HPV agreement between the two techniques was 95.85% (Table 2). When comparing the HPV results obtained with the Linear Array as the gold standard, the false-positive rate for general HPV and high-risk HPV genotyping Anyplex™ II HPV28 were 2.34% and 2.57%, respectively.

**Table 2:** Comparison of LA and Anyplex™ II HPV28 genotyping for overall and high-risk HPV positivity.

	Anyplex™ II HPV28	Linear Array	
		Positive	Negative
Overall HPV	Positive	1497 (87.60%)	40 (2.34)
	Negative	31 (1.81%)	141 (8.25%)
HR-HPV	Positive	1207 (70.63%)	44 (2.57%)
	Negative	108 (6.32%)	350 (20.48%)

The overall sensitivity and high-risk HPV sensitivity for Anyplex™ II HPV28, using Linear Array as the gold standard, exhibited similar values of 97% (95% CI, 96%–98%) and 96% (95% CI, 95%–97%), respectively. Considering type-specific HR-HPV, the sensitivity varied between 98% for HPV56 and 71% for HPV52. In the LR-HPV types, the sensitivity varied from 99% (95% CI, 95%–100%) for HPV42 to 25% (95% CI, 20%–32%) for HPV6. Specificity was 82% (95% CI, 75%–87%) for overall HPV and 76% (95% CI, 72%– 80%) for HR-HPV. Considering type-specific specificity, the majority was above 95%, the lowest value being observed for HPV42 (90%; 95% CI, 89%–92%) (Table 3).

**Table 3:** Prevalence of HPV genotypes detected in genital samples, genotype-specific agreement Anyplex^TM^ II HPV28 and LA and its specificity/sensitivity.

HPV Genotype	Linear Array n [% (95% CI)]	Anyplex^TM^ II HPV28 n [% (95% CI)]	Kappa κ (95% CI)	Sensitivity (95% CI)	Specificity (95% CI)
**Overall HPV**	1,577 [89.86 (88.44–91.27)]	1,529 [89.41 (87.96–0.87)]	78 (72–83)	97 (96–98)	82 (75–87)
**HR-HPV**	1,280 [72.93 (70.85–75.01)]	1,315 [76.91 (74.90–78.90)]	76 (73–80)	96 (95–97)	76 (72–80)
**6**	225 [12.8 (11.2–14.4)]	90 [5.3 (4.2–6.4)]	31 (24–38)	25 (20–32)	98 (97–98)
**11**	49 [2.8 (2.0–3.6)]	67 [3.9 (3.0–4.9)]	80 (71–87)	96 (86–99)	99 (98–99)
**16**	330 [18.8 (17.0–20.3)]	350 [20.5 (18.6–22.4)]	89 (86–92)	95 (92–97)	97 (96–98)
**18**	157 [8.9 (7.6–10.3)]	165 [9.7 (8.3–11.1)]	89 (85–93)	94 (89–97)	99 (98–99)
**26**	60 [3.4 (2.6–4.3)]	71 [4.2 (3.2–5.1)]	82 (75–89)	93 (83–98)	99 (98–99)
**31**	154 [8.8 (7.4–10.1)]	204 [12.0 (10.4–13.5)]	78 (73–83)	94 (89–97)	96 (95–97)
**33**	70 [4.0 (3.1–4.9)]	97 [5.7 (4.6–6.8)]	73 (66–81)	90 (80–96)	98 (97–99)
**35**	123 [7.0 (5.8–8.2)]	192 [11.3 (9.8–12.8)]	73 (67–79)	97 (93–99)	95 (94–96)
**39**	143 [8.1 (6.9–9.4)]	202 [11.8 (10.3–13.4)]	72 (67–78)	91 (85–95)	95 (94–96)
**40**	49 [2.8 (2.0–3.6)]	161 [9.5 (8.1–10.1)]	42 (33–50)	98 (89–100)	93 (92–94)
**42**	120 [6.8 (5.6–8.0)]	273 [16.1 (14.3–17.8)]	55 (50–61)	99 (95–100)	90 (89–92)
**45**	132 [7.5 (6.3–8.8)]	128 [7.5 (6.3–8.8)]	82 (77–87)	82 (75–89)	99 (98–99)
**51**	222 [12.6 (11.1–14.2)]	253 [14.8 (13.2–16.5)]	80 (76–84)	89 (85–93)	96 (95–97)
**52**	311 [17.7 (15.9–19.5)]	273 [16.0 (14.3–17.8)]	68 (64–73)	71 (65–76)	96 (94–97)
**53**	244 [14.0 (12.3–15.5)]	268 [15.8 (14.1–17.5)]	86 (82–89)	94 (90–97)	97 (96–98)
**54**	165 [9.4 (8.0–10.8)]	243 [14.3 (12.7–16.0)]	72 (67–77)	94 (90–97)	94 (93–95)
**56**	132 [7.52 (6.3–8.8)]	227 [13.3 (11.7–15.0)]	68 (62–74)	98 (93–100)	94 (92–95)
**58**	222 [12.6 (11.1–14.2)]	252 [14.8 (13.1–16.5)]	84 (80–88)	93 (89–96)	97 (96–97)
**59**	209 [11.9 (10.4–13.4)]	197 [11.6 (10.0–13.0)]	85 (80–89)	85 (79–89)	98 (98–99)
**61**	210 [12.0 (10.4–13.5)]	180 [10.1 (9.1–12.1)]	82 (78–86)	79 (73–84)	99 (98–99)
**66**	203 [11.6 (10.1–13.1)]	220 [12.9 (11.3–14.5)]	79 (74–83)	86 (80–90)	97 (96–98)
**68**	114 [6.5 (5.3–7.6)]	220 [12.9 (11.3–14.5)]	56 (49–62)	88 (81–94)	92 (91–94)
**69**	32 [1.8 (1.2–2.4)]	45 [2.6 (1.9–3.4)]	80 (70–90)	97 (84–100)	99 (99–100)
**70**	90 [5.1 (4.0–6.2)]	110 [6.5 (5.3–7.7)]	82 (78–88)	93 (86–97)	98 (98–99)
**73**	138 [7.9 (6.6–9.7)]	159 [9.4 (8.0–11.0)]	84 (79–89)	93 (87–96)	98 (97–99)
**82**	99 [5.6 (4.6–6.7)]	139 [8.2 (6.9–9.5)]	74 (68–81)	92 (85–96)	97 (96–98)

Cohen’s kappa score (κ) for most of the HPV types identified by the two tests was considered excellent (≥0.8), including the most prevalent HR-HPV types, HPV 16 and 18. Besides that, nine genotypes were considered to have good κ scores. The κ scores for three other types (HPV40, 42, and 68) were considered moderate, and only that of the HPV6 type was considered fair (Table 3).

**Fig 1:**
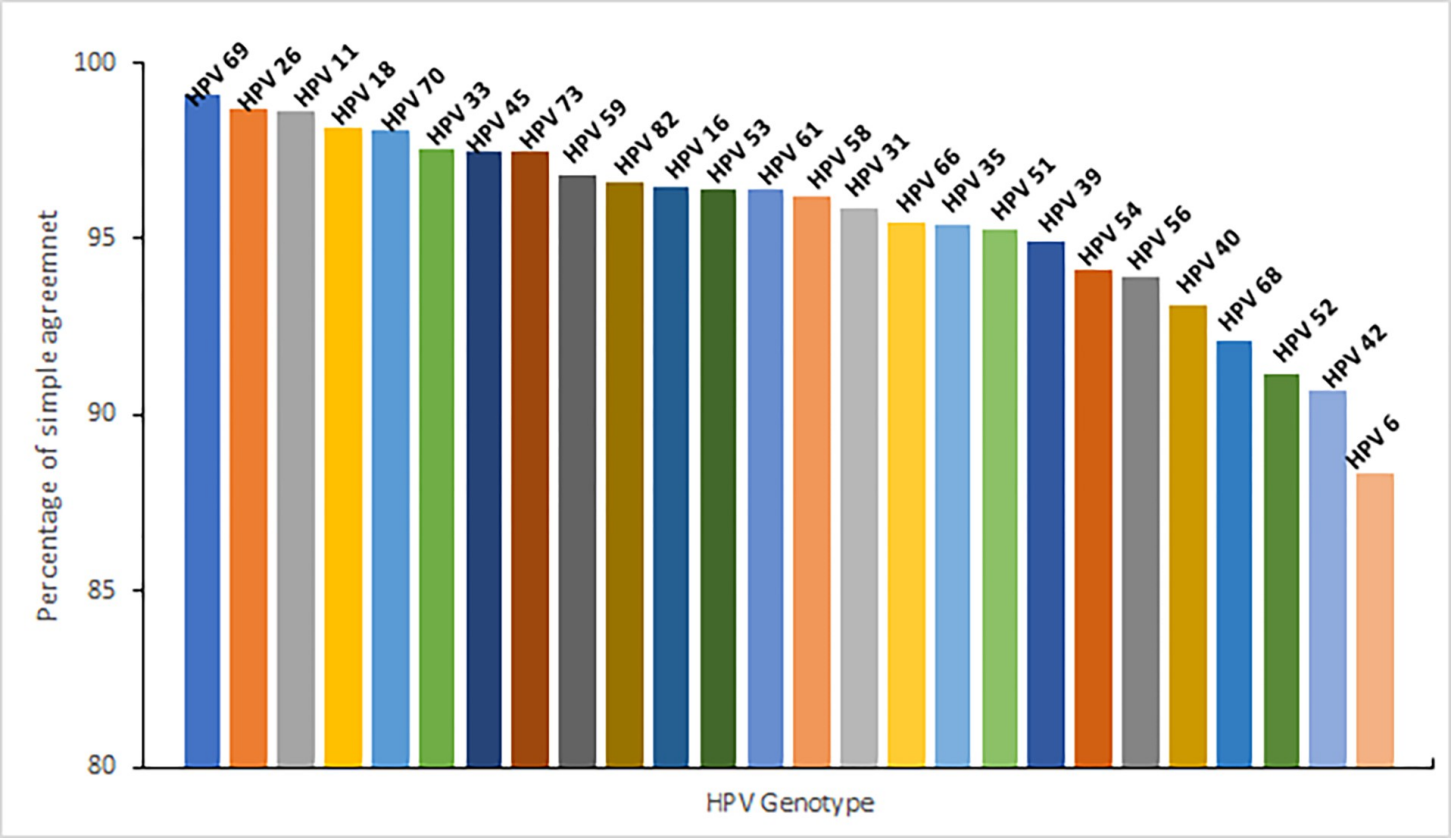
Percentage of agreement (positive and negative) for each individual HPV genotype comparing results obtained in LA and Anyplex™ II HPV28 assays.

The individual genotype agreement exhibited the highest percentage for HPV69 (99.12%), followed by HPV26 (98.71%) and HPV11 (98.64%). On the other hand, the lowest percentages were observed for HPV6 (88.37%), HPV42 (90.73%), and HPV52 (91.19%) (Fig 1).

**Fig 2:**
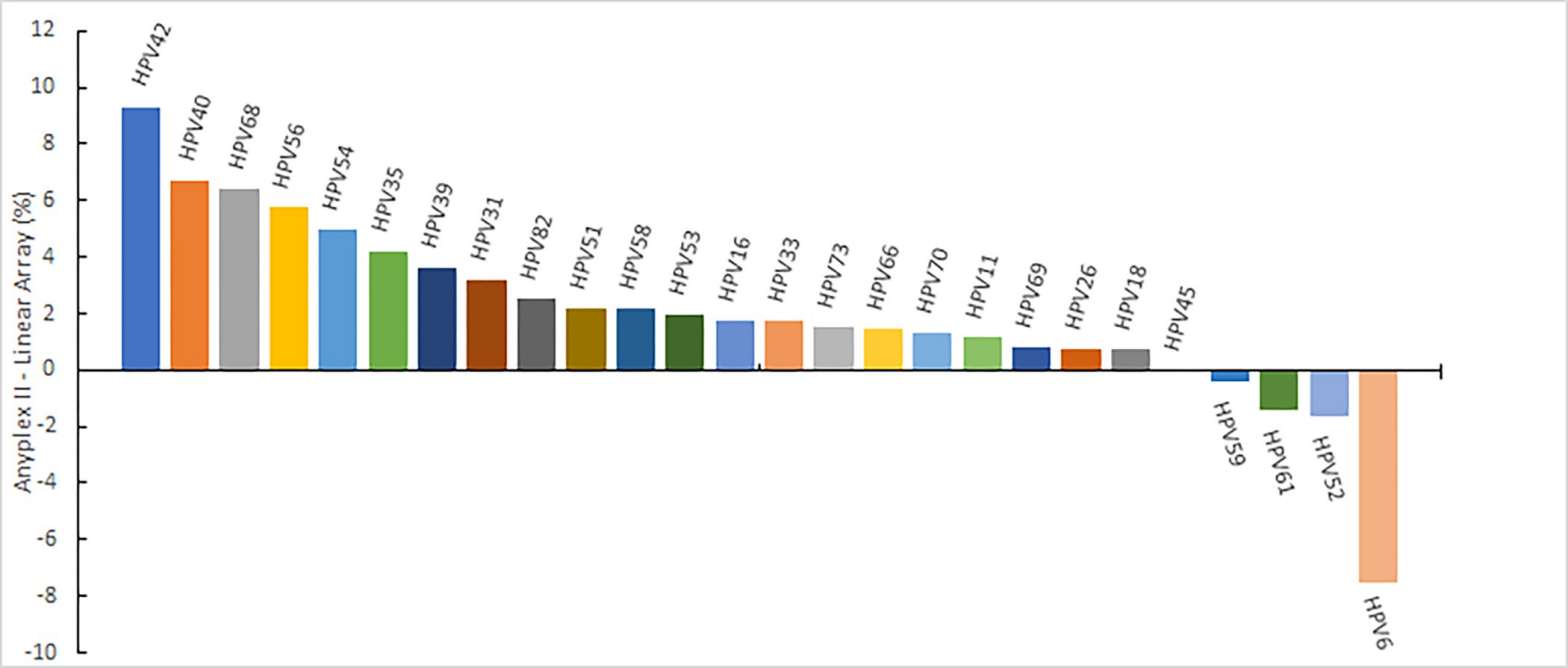
Difference in percentage HPV genotype positivity using Anyplex™ II and LA.

Among the 26 types analyzed individually, Anyplex™ II HPV28 detected 22 types more frequently, while LA exhibited higher detectability for HPV types 59, 61, 52, and 6. Fig 2 illustrates the difference in the percentage HPV genotypes positivity observed between the two methodologies.

Considering the percentage agreement in relation to genotype detection, HPV52 was the type that presented more false-negative results (62 samples, 3.64%). Conversely, HPV6 showed the highest number of false-positive results (162 samples, 9.56%). Table 4 presents the results regarding the most frequently detected genotypes by LA.

**Table 4:** Percentage agreement in relation to the genotypes most detected by the LA methodology.

	Anyplex™ II HPV28	Linear Array HPV Genotyping Test	
		Positive	Negative
**HPV59**	Positive	174 (10.22%)	31 (1.82%)
	Negative	23 (1.35%)	1475 (86.61%)
**HPV61**	Positive	163 (9.62%)	44 (2.60%)
	Negative	17 (1%)	1471 (86.78%)
**HPV52**	Positive	211 (12.39%)	88 (5.17%)
	Negative	62 (3.64%)	1342 (78.80%)
**HPV6**	Positive	55 (3.24%)	162 (9.56%)
	Negative	35 (2.06%)	1443 (85.13%)

## Discussion

Accurate measurement of HPV type prevalence over time is crucial for surveillance and evaluation of the implementation of public health strategies. Anyplex^**TM**^ II HPV28 has demonstrated acceptable reproducibility in detecting the HPV genotypes covered by the assays. Both tests exhibited comparable agreement and sensitivity, effectively detecting and identifying HPV types with no significant differences. LA, the gold standard methodology for HPV genotyping, has been widely used in clinical diagnosis and epidemiological research. However, since LA has been discontinued, the assessment of genotype prevalence over time presents a new challenge, while maintaining the necessary confidence to compare populations across different time periods. In several studies that compared HPV genotyping methodologies, Anyplex™ II HPV28 demonstrated good agreement with other HPV assays, including LA. This is likely due to its highly sensitive multiplexed detection approach [8, 28–30].

In the context of primary cancer screening, the majority of HPV screening tests typically involve 14 high-risk HPV types with partial genotyping, categorizing HPV 16, HPV 18, and a collective pool labeled as "others". However, both comprehensive global meta-analyses and extensive long-term cohort studies have consistently identified variations in the oncogenic potential among different HPV types. Notably, a Swedish study revealed that HPV types 16, 18, 31, 33, 45, or 52 were detected in 689 out of 808 screen-detected invasive cervical cancers (ICC), constituting 85.3% of cases [31]. The inclusion of additional HPV types, specifically 35, 39, 51, 56, 58, 59, 66, or 68, which are also encompassed in currently employed HPV tests, resulted in a minimal increase in prevalence, accounting for only 12 of the 808 cases (1.5% for all eight types collectively). Our study revealed that Anyplex™ II HPV28 exhibits a lower detection sensitivity for the low-risk genotype, HPV6. Cornall and colleagues (2017b) evaluated the HPV genotyping performance of two genotyping assays, Anyplex^**TM**^ II HPV28 (Seegene) and EuroArray HPV (EuroImmun), in comparison with LA. The agreement between LA and the other two assays was nearly perfect for most genotypes (κ = 0.81−1.00), including all high-risk genotypes (κ = 0.81–0.98). When Anyplex™ II was compared with LA, the prevalence of any of the 25 common HPV genotypes was slightly higher in LA (86.5%) compared to Anyplex™ II HPV28 (85.4%), but this difference was not statistically significant (p = 0.326). Additionally, the authors reported that Anyplex™ II demonstrated similar sensitivity to LA for most high-risk genotypes, with slightly lower sensitivity observed for HPV51 or 52 [14, 32]. As suggested by Sundstrom (2021) and Nygard (2021), HPV screening tests in the post-vaccination era may exhibit enhanced performance if restricted to the HPV types covered by the nonavalent vaccine. Moreover, the consideration of screening for all 14 HPV types could potentially lead to a suboptimal balance between the associated harms and benefits [31, 33].

The LA assay has a limitation in comparison with Anyplex™ II HPV28, which is the cross-reactivity of the HPV52 probe with HPV33, 35, and 58. This requires the use of an additional PCR test to confirm the status of HPV52, increasing the complexity and cost of the method. In our study, LA detected more cases of HPV52 than Anyplex™ II HPV28, with a substantial Kappa score of 0.68. It is important to note that subjecting the samples to defrosting could potentially increase the degradation of the genetic material. However, it is worth mentioning that this does not significantly impact the sensitivity of the assays [34, 35].

## Conclusions

The study findings reveal that both the Linear Array (LA) and Anyplex™ II HPV28 assays exhibited robust agreement in HPV detection and in high-risk typing from genital samples. The Seegene assay, in particular, demonstrated excellent performance as an HPV screening tool in epidemiological studies. Its notable concordance with the Linear Array suggests its reliability, positioning it as a viable alternative to other commercially available HPV diagnostic tests. These results underscore the potential of the Anyplex™ II HPV28 assay for effective and accurate HPV screening in diverse research applications.
